# Exploring the Thermal-Oxidative Stability of Azithromycin Using a Thermoactivated Sensor Based on Cerium Molybdate and Multi-Walled Carbon Nanotubes

**DOI:** 10.3390/nano14110899

**Published:** 2024-05-21

**Authors:** Heryka R. A. Costa, André O. Santos, Yago N. Teixeira, Maria A. S. Silva, Valker A. Feitosa, Simone Morais, Thiago M. B. F. Oliveira

**Affiliations:** 1 Centro de Ciência e Tecnologia, Universidade Federal do Cariri, Av. Tenente Raimundo Rocha, 1639, Cidade Universitária, Juazeiro do Norte 63048-080, CE, Brazil; heryka.abrantes@aluno.ufca.edu.br (H.R.A.C.); andre.oliveira@ufca.edu.br (A.O.S.); yago.neco@aluno.ufca.edu.br (Y.N.T.); aparecida.santiago@ufca.edu.br (M.A.S.S.); 2 Departamento de Tecnologia Bioquímico-Farmacêutica, Universidade de São Paulo, Av. Prof. Lineu Prestes, 580, Butantã, São Paulo 05508-000, SP, Brazil; valker@usp.br; 3 REQUIMTE–LAQV, Instituto Superior de Engenharia do Porto, Instituto Politécnico do Porto, Rua Dr. Bernardino de Almeida 431, Porto 4249-015, Portugal; sbm@isep.ipp.pt

**Keywords:** azithromycin, thermal-oxidative stability, cerium molybdate, carbon nanotubes, composite materials, electrochemical sensor

## Abstract

The chemical stability of azithromycin (AZM) may be compromised depending on the imposed thermo-oxidative conditions. This report addresses evidence of this process under varying conditions of temperature (20–80 °C), exposure time to UV radiation (1–3 h irradiation at 257 nm), and air saturation (1–3 h saturation with atmospheric air at 1.2 L min^−1^ and 15 kPa) through electrochemical measurements performed with a thermoactivated cerium molybdate (Ce_2_(MoO_4_)_3_)/multi-walled carbon nanotubes (MWCNT)-based composite electrode. Thermal treatment at 120 °C led to coordinated water elimination in Ce_2_(MoO_4_)_3_, improving its electrocatalytic effect on antibiotic oxidation, while MWCNT were essential to reduce the charge-transfer resistance and promote signal amplification. Theoretical–experimental data revealed remarkable reactivity for the irreversible oxidation of AZM on the working sensor using phosphate buffer (pH = 8) prepared in CH_3_OH/H_2_O (10:90%, *v*/*v*). Highly sensitive (230 nM detection limit) and precise (RSD < 4.0%) measurements were recorded under these conditions. The results also showed that AZM reduces its half-life as the temperature, exposure time to UV radiation, and air saturation increase. This fact reinforces the need for continuous quality control of AZM-based pharmaceuticals, using conditions closer to those observed during their transport and storage, reducing impacts on consumers’ health.

## 1. Introduction

Macrolide antibiotics are characterized by a macrocyclic lactone ring structure—12 to 16 atoms—with one or more sugar chains attached [1,2,3]. They are bacteriostatic agents against Gram-positive (*Streptococcus pneumoniae*, *Streptococcus pyogenes*, *Streptococcus agalactiae*, *Staphylococcus aureus*, and *Corynebacterium diphtheriae*) and Gram-negative microorganisms (*Haemophilus influenzae* and *Moraxella catarrhalis*), hindering their growth and reproduction by interfering with ribosome function and inhibiting protein synthesis [1]. Macrolides are often prescribed to treat respiratory tract and skin infections, sexually transmitted diseases, and various other bacterial infections [2]. Azithromycin (AZM; 9-deoxy-9*α*-aza-9*α*-methyl-9*α*-homoerythromycin A) is among the most prescribed second-generation macrolides, standing out for its broader spectrum of activity and improved pharmacokinetic properties in relation to erythromycin, i.e., its first-generation precursor [3]. To guarantee effectiveness, AZM-based pharmaceutical compositions require special care to guarantee the stability of the active ingredient under ideal thermal-oxidative conditions [4,5]. Unfortunately, these recommendations are not always followed during the transport and/or storage of pharmaceutical products [6], demanding more rigorous quality control to determine the antibiotic’s half-life in atypical circumstances and ensure its effectiveness.

Liquid chromatography achieves proper resolution to analyze AZM alongside excipients, justifying its broad acceptance in studies with bulk samples and formulations [6]. The main limitations are expensive columns, unstable detectors, and high pH and/or temperature conditions to use traditional silica-based columns [6,7]. There are also reports based on microbiology [8], spectroscopy [9], capillary electrophoresis [10], and electroanalysis [11]. All have analytical credibility, but the advantages found with electroanalytical methods in terms of operation, specificity, precision, robustness, possibility of miniaturization, and portability, along with the reduction of waste generated in the laboratory routine, make them highly attractive.

The success of electroanalytical methods depends on the design of the sensors and, consequently, the electrode materials used as the reaction interface. Focusing on robust devices, carbon nanotubes stand out among the most used materials, given the rigidity of C–C bonds even at high pressures and impressive surface-to-volume ratio, in addition to having charge-transport-inducing structural defects distributed throughout their crystalline nanostructures [12]. The integration of carbon nanotubes with advanced ceramics has also attracted great scientific interest, as it results in composites with electrochemical properties superior to their counterparts [13,14,15]. Cerium (III) molybdate, Ce_2_(MoO_4_)_3_, for example, is a rare-earth-based electroceramic material with extraordinary chemical stability, even under critical pH [16], temperature [17,18], and pressure conditions [19,20]. Furthermore, the cerium present in the unit cell can assume different oxidation states, positively influencing the energy density, charge–discharge time, and (photo)electrochemical reactivity [21]. These features also justify the increasing use of this material in the construction of sensors [21,22], catalysts [23,24,25], energy conversion devices [26,27], batteries [26,28], and capacitors [29], among other emerging technologies.

For electrochemical sensors, there are still few reports proving the viability of composite materials based on carbon allotropes and rare-earth molybdates, and there is no evidence of application for antibiotic electroanalysis. This article fills part of this gap, presenting an electrochemical sensor built with a Ce_2_(MoO_4_)_3_/multi-walled carbon nanotubes (MWCNT) composite, which demonstrated high performance for monitoring AZM under different thermal-oxidative conditions caused by temperature, exposure to UV radiation, and saturation with atmospheric air. The data found raise awareness about the importance of appropriate storage of pharmaceutical formulations.

## 2. Materials and Methods

### 2.1. Chemicals

The AZM standard was kindly provided by Indústria Farmacêutica Cristália (Itapita—SP, Brazil). The AZM stock solution was prepared in methanol/water (CH_3_OH/H_2_O; 50:50%; *v*/*v*) (Sigma-Aldrich, São Paulo, Brazil). The potassium ferrocyanide (K_4_Fe(CN)_6_) redox probe, as well as the synthesis reagents, including cerium nitrate (Ce(NO_3_)_3_·6H_2_O), ammonium molybdate ((NH_4_)_6_Mo_7_O_24_·4H_2_O), ethylenediamine tetraacetic acid (EDTA), and ammonium hydroxide (NH_4_OH), were also obtained from Sigma-Aldrich and used without further purification. Sodium mono-(Na_2_HPO_4_) and di-hydrogen phosphate (NaH_2_PO_4_) (both from Vetec, Rio de Janeiro, Brazil) were used to prepare the buffered electrolyte in CH_3_OH/H_2_O (10:90%; *v*/*v*). Phosphoric acid (H_3_PO_4_) and sodium hydroxide (NaOH) solutions at 0.01 M were used for pH adjustments, with reagents purchased from Dinâmica, São Paulo, Brazil. Ultra-purified water (*ρ* = 18 MΩ cm^−1^) was used to prepare all aqueous solutions. The MWCNT (10 nm diameter and 1.5 μm average length) used for electrode modification were obtained from DropSens, Oviedo, Spain.

### 2.2. Cerium Molybdate Synthesis

The production of Ce_2_(MoO_4_)_3_ was based on the report by Gomes et al. [21]. Summarizing the synthesis route followed, EDTA was dissolved in NH_4_OH (1 g:10 mL) and the resulting mixture was used as a dilution medium for solutions of Ce(NO_3_)_3_·6H_2_O and (NH_4_)_6_Mo_7_O_24_·4H_2_O, both prepared at 1.0 μM. Then, 25 mL of each solution was transferred into a Teflon autoclave, which was sealed and kept at 423 K for 6 h (5 K min^−1^ heating rate). After cooling, the yellow crystalline precipitate of Ce_2_(MoO_4_)_3_ was decanted, washed several times with deionized water, and finally dried at 333 K for 24 h.

### 2.3. Electrochemical Sensor Development

A graphite and paraffin-based carbon paste (70:30%, *w*/*w*) was used as a dispersion matrix, after complete homogenization of the precursors in an agate mortar. Then, Ce_2_(MoO_4_)_3_ (5%; *w*/*w*) and MWCNT (10%; *w*/*w*) were added, followed by homogenization and, when necessary, thermoactivation of the resulting composite at 373 K for 1 h. This material was inserted into a Teflon^®^ tube (1.0 mm internal diameter) containing a 1020 stainless steel piston for electrical contact. The resulting sensor was defined as Ce_2_(MoO_4_)_3_/MWCNT-CPE and, before using it, its surface was smoothed against low-roughness bond paper. To compare results, working electrodes were also constructed with unmodified carbon paste (CPE) and modified with carbon nanotubes (MWCNT-CPE) or molybdate (Ce_2_(MoO_4_)_3_-CPE).

### 2.4. Physicochemical Characterization

Different techniques were explored to understand the physicochemical properties of electrode materials in terms of morphology, composition, and crystallinity, including scanning electron microscopy (SEM; Tescan Vega 3, Tokyo, Japan), performed with a 30 keV electron beam; Raman spectroscopy (Micro-Raman Senterra, Bruker, Ettlingen, Germany), using a solid-state laser that operated at 532 nm with 5 mW power; X-ray diffraction (XRD; Bruker D8 Advance XRD diffractometer, Ettlingen, Germany), using CuKα radiation (λ = 1.5418 Å) in the 2θ range from 10° to 90°; Fourier-transform infrared spectroscopy (FTIR; Cary 630, Agilent, Santa Clara, CA, USA); and thermogravimetric analysis (TGA—50, Shimadzu, Kyoto, Japan). Electrochemical data were obtained by voltammetric and impedimetric experiments, using a modular potentiostat/galvanostat (PGSTAT 128 N/FRA32, Metrohm-Autolab, Utrecht, The Netherlands).

### 2.5. Computer Simulations

The factors influencing AZM solubility in H_2_O, CH_3_OH, and CH_3_OH/H_2_O combinations were evaluated by theoretical experiments involving semi-empirical and quantum calculations. The GROMACS software (https://www.gromacs.org/) was used for molecular dynamics studies, while the solvent approximation parameters were evaluated by the MDAnalysis package (https://www.mdanalysis.org/). The tests were performed based on cubic boxes designed by the Packmol software (https://m3g.github.io/packmol/), with an initial edge size of 4.7 nm, and filled with AZM and solvent molecules in proportions sufficient to approximate the mixture density (*ρ*; g cm^−3^) closer to that observed at 293 K: *ρ*_(H2O)_ = 0.99705; *ρ*_(CH3OH)_ = 0.79140; *ρ*_(10% CH3OH)_ = 0.97159; *ρ*_(20% CH3OH)_ = 0.94980; *ρ*_(30% CH3OH)_ = 0.92845; *ρ*_(40% CH3OH)_ = 0.90658; and *ρ*_(20% CH3OH)_ = 0.88557. The data obtained were considered in order to understand the distribution of solvent molecules around the AZM molecule (i.e., radial distribution), as well as the preferred conformation of the atoms, evaluated in terms of root mean square fluctuation (RMSF).

### 2.6. Electroanalytical Measurements and Performance

Electrochemical data acquisition was performed at 25 °C using a conventional three-electrode cell: (i) CPE, MWCNT-CPE, Ce_2_(MoO_4_)_3_-CPE, or Ce_2_(MoO_4_)_3_/MWCNT-CPE as a working electrode; (ii) platinum rod as a counter electrode; and (iii) Ag/AgCl as a reference electrode. Qualitative information on redox reaction mechanism, mass- and charge-transport processes, electrode modification, and analyte stability under thermal-oxidative conditions were analyzed by Cyclic Voltammetry (CV; 25–250 mV s^−1^) and Electrochemical Impedance Spectroscopy (EIS; 100 MHz to 100 kHz frequency range; and 5 mV modulation amplitude).

To quantify AZM (1.19–22.0 µmol L^−1^), Square Wave Voltammetry (SWV) was used after finding the best electroanalytical conditions in terms of pulse frequency (*f*; 10–300 Hz), amplitude (5–50 mV), and potential increment (1–7 mV). The choice of the optimum values for each variable was based on the signal-to-noise ratio, voltammetric profile, peak current (*I_p_*), peak potential (*E_p_*), and half-peak width (*E_p/_*_2_). Limits of detection (LOD) and quantification (LOQ) were determined using linear regression coefficients obtained from the analytical curve [30]. To assess the precision of the proposed procedure, typical intraday (*n* = 10) and interday (*n* = 5) measurements of 10 µM AZM were performed with a single sensor, considering the relative standard deviation of the results (RSD; %). Reproducibility with different devices (*n* = 3) was also tested under the same experimental conditions. Excipients conventionally used in AZM-based pharmaceutical formulations (lactose, polycaprolactone, poloxamer, and soy phospholipid) were tested as potential interferers. Results obtained from the electroanalytical procedure were also validated by UV-Visible spectrophotometry performed at 547 nm [31], with the data expressed as the arithmetic mean between triplicates.

### 2.7. Studies on AZM’s Thermal-Oxidative Stability

Given the suspicion of accelerated degradation of AZM under more critical thermal-oxidative conditions, the electroanalytical method developed with Ce_2_(MoO_4_)_3_/MWCNT-CPE was tested to evaluate the stability of the aforementioned antibiotic under varying conditions of temperature (20–80 °C), exposure to ultraviolet light (1–3 h irradiation at 257 nm), and saturation with atmospheric air (1–3 h bubbling atmospheric air at 1.2 L min^−1^ and 15 kPa). The influence of these parameters was evaluated separately and combined. In the latter case, the effect on the accelerated degradation of AZM was studied using a 2^3^ full factorial experimental design and response surface methodology (RSM) [32,33]. In total, 15 experiments were performed (12 trials and 3 central points) and the degradation percentages, estimated by the decrease in compound concentration, were obtained by the following polynomial equation model:

(1) $$Y=\beta_{0}+\overset{n}{\underset{i=1}{\sum }}\beta_{i}x_{i}+\overset{n}{\underset{i=1}{\sum }}\beta_{ii}x_{i}^{2}+\overset{n-1}{\underset{i=1}{\sum }}\overset{n}{\underset{j=i+1}{\sum }}\beta_{ij}x_{i}x_{j}$$

where Y is the percentage of AZM degradation; $\beta_{0}$ represents the intercept coefficient; $\beta_{i}$, $\beta_{ii}$, and $\beta_{ij}$ are regression coefficients; and $x_{i}$ and $x_{j}$ are the independently studied variables. The predicted and observed values in each condition are shown in Table S1—Supplementary Materials.

## 3. Results and Discussion

### 3.1. Relationship between AZM Solubility and Electroactivity

**Figure 1:**
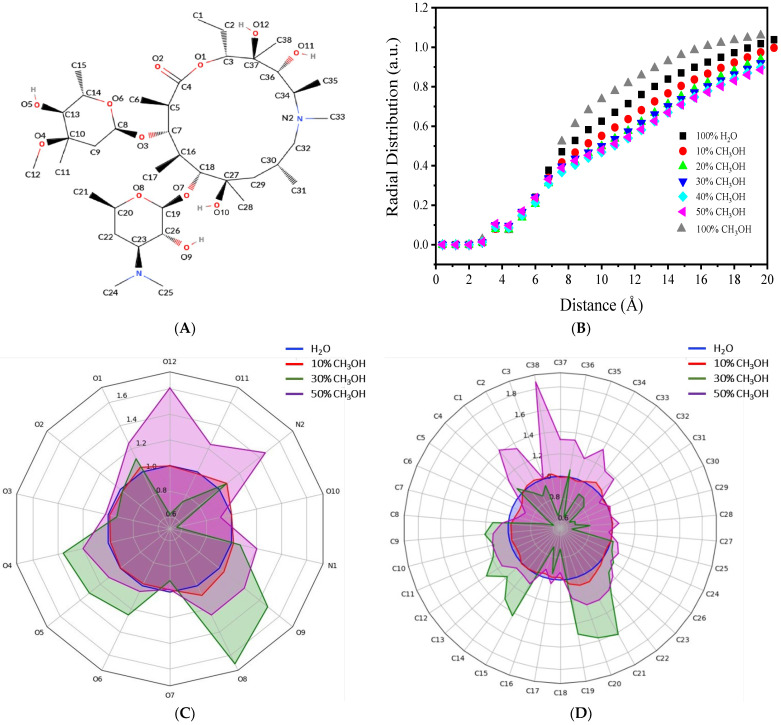
(**A**) AZM chemical structure (numbering for illustration only), followed by (**B**) radial distribution of H_2_O and CH_3_OH around the antibiotic molecules. Root mean square fluctuation of (**C**) oxygen/nitrogen and (**D**) carbon atoms in the AZM structure at different proportions of H_2_O and CH_3_OH.

The AZM molecule is formed by three interconnected cyclic structures composed mainly of carbon atoms (Figure 1A), with low solubility expected in purely aqueous matrices, although there are nitrogen and oxygen sites available for hydrogen bonds. Therefore, different combinations of CH_3_OH:H_2_O were evaluated to identify the most suitable solvent to solubilize the analyte and allow the correct assessment of its electroactivity. With this goal in mind, theoretical experiments were performed based on semi-empirical and quantum calculations that explain the radial distribution of solvents around the solute. The results indicated that H_2_O forms solvation layers more than 20 Å away from the solute (radial distribution ≈ 1.0; Figure 1B), although the most effective interactions for solubilization do not exceed 5 Å [34], since hydrogen bonds have a directional character, requiring that the donor and acceptor groups are properly aligned and in close contact. As the AZM–H_2_O interaction occurs through a limited number of reactive sites, the solubility of the antibiotic is impaired and makes its electrochemical characterization difficult due to the low concentration available in the redox equilibrium.

The relationship between radial distribution and solute distance improves with CH_3_OH, possibly due to the greater abundance of C–C interactions in this system. However, concentrated CH_3_OH has a high evaporation rate and compromises the electrode materials by erosion, jeopardizing its choice as an ideal solvent. CH_3_OH:H_2_O combinations are easier to manipulate, enabling polarity control as the alcohol concentration increases from 10% to 50% (*v*/*v*). This process also reduces solute solvation by solvent molecules more than 5 Å apart, possibly displaced by hydrophobic repulsion between the components of the CH_3_OH:H_2_O mixture. Another interesting point is that the addition of CH_3_OH almost does not influence the distribution of solvation layers closer to the solute, as well as contributes to solubilizing it through dipole-induced dipole attraction and London dispersion forces.

Solute–solvent interactions are also directly related to the exposure/conformation of atoms that participate in intramolecular and intermolecular interactions. In this work, this information was evaluated in terms of RMSF; higher values of this variable indicate greater susceptibility of the solute to solubilize [35]. In Figure 1C,D, the RMSF values increase from the center towards the limit of the circumferences. In Figure 1C, when there is only H_2_O in the system, the O and N atoms of the AZM molecule are less available for interactions, making their solubilization by hydrogen bonds difficult. This aspect changes in the presence of CH_3_OH, especially using 50% (*v*/*v*) of this solvent, which increases the amount and intensity in which the N and O atoms of the rings become more accessible to the solvent. This behavior occurs even in the largest ring of the structure, which is mainly responsible for its hydrophobicity.

The benefits of the CH_3_OH/H_2_O combination also extend to carbon atoms (Figure 1D), as the presence of alcohol favors the emergence of new interactions. This mixture also triggers changes in dielectric constant (*ε_r_* ≈ 50 for 50% CH_3_OH, *v*/*v*) compared to the isolated solvents (*ε_r_* = 78.36 for H_2_O and *ε_r_* = 32.61 for CH_3_OH), changing the conformational stability of the carbon atoms that bind to the sugar rings (C7 and C18) and influence the solubility of the AZM molecule. Given these results, the AZM stock solution was prepared with a mixture of CH_3_OH:H_2_O (50:50% *v*/*v*), corroborating data published by Cao et al. [36], who found greater solubility for AZM in solvents of intermediate polarity.

**Figure 2:**
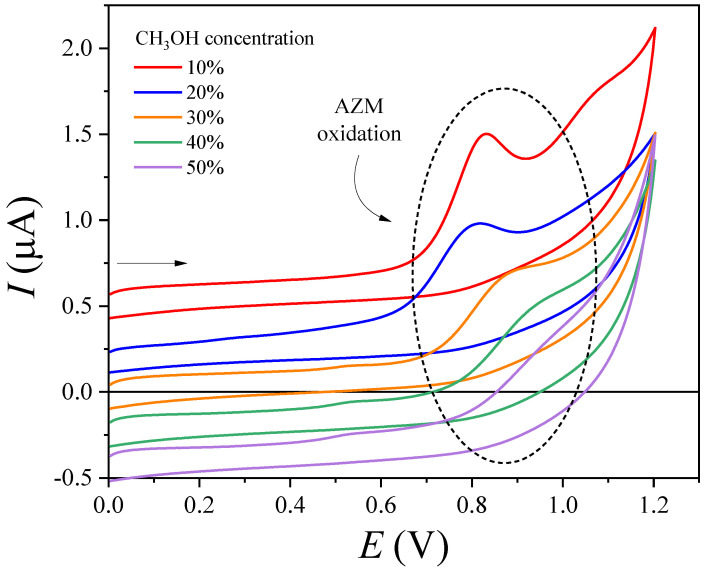
Cyclic voltammograms (first cycle) recorded for 10 µM AZM at 50 mV s^−1^, using Ce_2_(MoO_4_)_3_/MWCNT-CPE as an electrochemical sensor, immersed in 1.0 mM phosphate buffer (pH = 8.0) prepared in different CH_3_OH/H_2_O combinations.

CH_3_OH was also used in the supporting electrolyte. From Figure 2, it is observed that the presence of this alcohol also affects the intensity, potential, and voltammetric profile recorded for AZM oxidation. Keeping the analyte concentration at 10 µM in electrolytes with [CH_3_OH] > 10% (*v*/*v*), there was a loss of resolution, reduction in *I_p_* values, and increase in *E_p_* values, caused by increased viscosity and mass transport limitation at the electrode/solution interface. The electroanalytical signal also decreased with [CH_3_OH] < 10% (*v*/*v*) due to low solubility and availability of the analyte in the electrochemical cell. Given these results, phosphate buffer prepared in a binary mixture of CH_3_OH/H_2_O (10:90%, *v*/*v*) was used as an electrolyte in the following experiments.

### 3.2. Characterization of Electrode Materials

**Figure 3:**
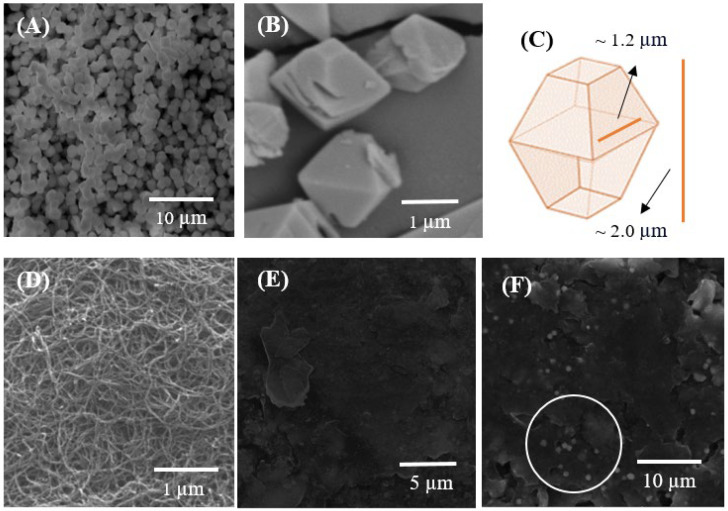
SEM images for Ce_2_(MoO_4_)_3_ after (**A**) 5000× and (**B**) 10,000× magnification, complemented by a (**C**) theoretical projection of the microparticles produced. The results obtained for (**D**) MWCNT, (**E**) MWCNT-CPE (10:90%, *w*/*w*), and (**F**) Ce_2_(MoO_4_)_3_-MWCNT-CPE (30:10:60%, *w*/*w*/*w*) were recorded after 1000× magnification.

The physicochemical features of the electrode materials were evaluated regarding morphology, crystallinity, and composition. Starting with the morphological aspects, the micrograph in Figure 3A proves that the as-prepared Ce_2_(MoO_4_)_3_ has characteristics of polydisperse microparticles, prone to agglomeration by an Ostwald Ripening-type mechanism (total energy reduction by increasing system scale size), which is common for materials with high surface charge. Observing molybdate at a higher magnification level (Figure 3B), microstructures shaped like truncated octahedra can be seen. Figure 3C shows a theoretical projection of the Ce_2_(MoO_4_)_3_ produced, with ~2.0 µm in the cross section and ~1.2 µm in the prismatic plane base. MWCNT tangles are also visible before combining them into the carbon composite (Figure 3D) but become imperceptible after homogenization in the conductive mixture (Figure 3E). Ce_2_(MoO_4_)_3_ microparticles are more noticeable in proportions > 20% (*w*/*w*), appearing as non-agglutinated and randomly distributed microcrystals, highlighted in the white circle in Figure 3F. This feature is interesting for electrochemical sensors because it allows better use of the material’s semiconductor properties when compared to its agglomerated form.

**Figure 4:**
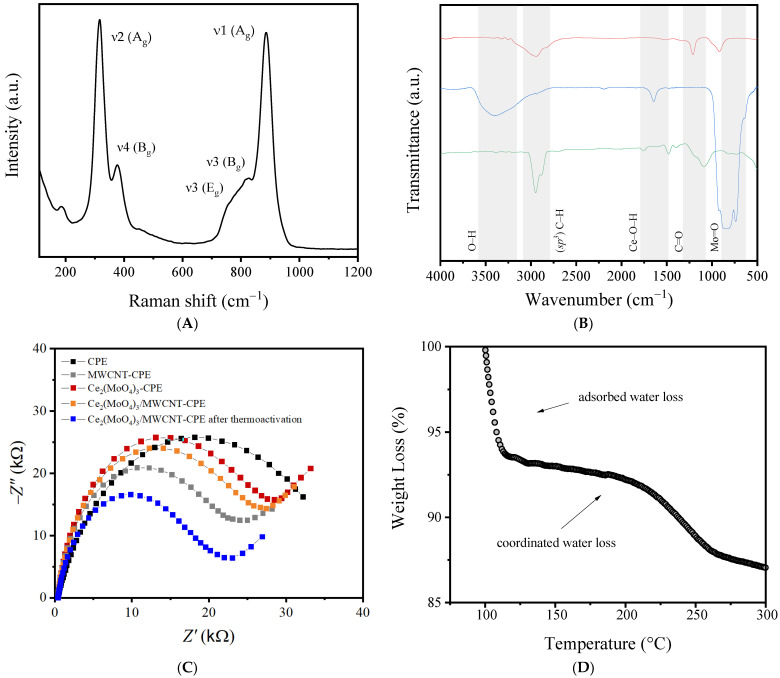
(**A**) Raman spectrum of Ce_2_(MoO_4_)_3_ used to develop the electrochemical sensor. (**B**) FTIR spectra recorded for MWCNT-CPE (red line), Ce_2_(MoO_4_)_3_ (blue line), and Ce_2_(MoO_4_)_3_/MWCNT-CPE (green line). (**C**) Nyquist diagrams obtained by electrochemical impedance spectroscopy for different electrode materials, using 0.1 mM K_3_[Fe(CN)_6_] as a redox probe and a frequency range between 10 kHz and 0.1 Hz. (**D**) Thermogravimetric analysis performed for the as-produced Ce_2_(MoO_4_)_3_.

The success of molybdate synthesis was also verified by Raman spectroscopy. The spectrum illustrated in Figure 4A reveals peaks between 670 and 1000 cm^−1^, attributed to symmetric and asymmetric Mo-O stretching vibrations; ν3 (E_g_), ν3 (B_g_), and ν1 (A_g_). Those between 250 and 500 cm^−1^ represent symmetric and asymmetric O–Mo–O deformations; ν2 (A_g_) and ν4 (B_g_) [20]. The signals below 220 cm^−1^ correspond to the transduction and libration processes of MoO_4_^2−^ and Ce^3+^ ions. The internal vibrational mode, with a sharp and intense profile at approximately 890 cm^−1^, indicates that the molybdate crystals are morphologically organized in a short range [21]. Additional XRD analysis of molybdate (Figure S1) indicated a remarkable structural organization and crystalline arrangement, characteristic of a tetragonal scheelite-like structure with space group *C*^6^_4*h*_ (*I*4_1_*/a*) (ICSD card No. 423509). The lattice parameters and quality indicators obtained by Rietveld refinement are available in Table S2. The FTIR spectrum for Ce_2_(MoO_4_)_3_ shows vibrational modes characteristic of the molybdate group (Figure 4B), highlighting a set of peaks between 950 cm^−1^ and 720 cm^−1^, resulting from terminal Mo=O stretching vibrations [23]. Vibrational modes ≥ 850 cm^−1^ are seen in tetragonal crystalline scheelite-type structures [37]. The sharp and lower intensity peak at 1450 cm^−1^ comes from Ce–O–H bending modes [38]. The broad band between 3600 cm^−1^ and 3100 cm^−1^ is associated with the O–H stretching and bending modes, possibly arising from reticular/interstitial water present in the structures. After dispersing the molybdate in the composite, the peaks related to carbon allotropes appear with greater intensity, as they are in superior proportion. The peaks between 3000 cm^−1^ and 2840 cm^−1^ refer to the C–H vibrational modes of *sp*^3^-hybridized carbon atoms. The existence of oxygenated functional groups in the MWCNT structure was also proven by C=O stretching vibrations around 1500 cm^−1^ [39].

Regarding the variations in charge-transfer resistance (*R_ct_*) caused by the combined materials, the information was assessed by EIS using 1.0 mM K_4_[Fe(CN)_6_] as a redox probe to monitor interfacial processes at 0.25 V. Based on Nyquist diagrams (Figure 4C), the system assembled with CPE has the highest capacitance, reaching *R_ct_* = 37.17 kΩ. For amperometric sensors, high *R_ct_* values impair the sensitivity at which the electroanalytical signal is recorded. In this sense, the addition of MWCNT is decisive for better electroanalysis performance, enabling the system to operate with *R_ct_* = 24.48 kΩ and, in this circumstance, facilitating the participation of diffusion processes in the electrical circuit. After adding Ce_2_(MoO_4_)_3_ to the composite, there is a further increase in the semicircle diameter, both in the presence (*R_ct_* = 29.67 kΩ) and absence of nanotubes (*R_ct_* = 31.91 kΩ), suggesting a new increase in electrical resistance. Although the molybdate produced is a semiconductor, the presence of water adsorbed and/or coordinated to the microcrystals can increase the charge-transfer resistance [40]. This hypothesis is confirmed by the thermogravimetric analysis in Figure 4D, where mass losses in the compound are recorded for both conditions. For this reason, Ce_2_(MoO_4_)_3_/MWCNT-CPE was thermoactivated at 120 °C for 1 h before electrochemical measurements, leading to a pronounced decrease in the system’s impedance (*R_ct_* = 18.44 kΩ) caused by water loss.

### 3.3. AZM Electroactivity

**Figure 5:**
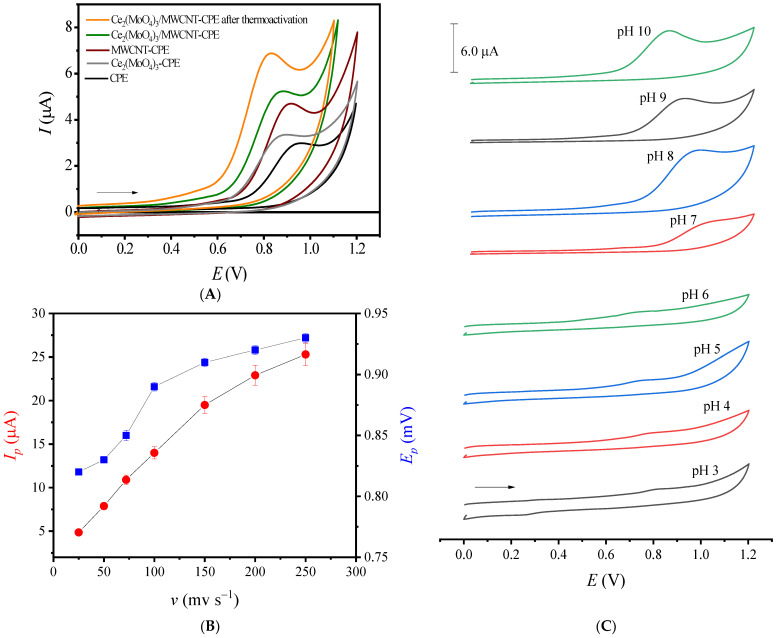
(**A**) Cyclic voltammograms (first cycle) recorded for 10 µM AZM at 50 mV s^−1^, using different electrochemical sensors, immersed in 1.0 mM phosphate buffer (pH = 8.0). (**B**) Influence of scan rate on peak currents and peak potentials, recorded during AZM oxidation on the thermoactivated Ce_2_(MoO_4_)_3_/MWCNT-CPE sensor. (**C**) Influence of pH on electrochemical behavior of AZM on the thermoactivated Ce_2_(MoO_4_)_3_/MWCNT-CPE sensor.

The reactivity of the different electrode materials for AZM oxidation was evaluated by CV at 50 mV s^−1^, using 1.0 mM phosphate buffer (pH = 8.0) prepared in CH_3_OH/H_2_O (10:90%, *v*/*v*) as an electrolyte. Figure 5A reveals a relatively low anodic process at 0.9 V when the redox reaction is studied on CPE, but this same electrochemical event is significantly amplified using MWCNT-CPE (1.81 µA increment in faradaic current), proving the importance of carbon nanotubes to improve device sensitivity. Among other advantages, MWCNT collectively provide greater active area, conductivity, and electronic transport kinetics for the redox reaction under study. Molybdate demonstrates a greater effect on the electrocatalysis of the redox reaction (oxidation peak 100 mV less positive), but also contributes to signal intensity after Ce_2_(MoO_4_)_3_/MWCNT-CPE thermoactivation, since the electrical resistivity is lower. A comparison of voltammetric signals recorded in the absence and presence of AZM can be seen in Figure S2—Supplementary Materials. The voltammograms recorded in the absence of the analyte were similar for all sensors tested. It is worth mentioning that no significant variation in the AZM oxidation current was observed even after fifty potential scans, confirming device stability.

The *I_p_* values generated by the drug oxidation progressively decreased after consecutive potential cycles, with *E_p_* also shifting towards more positive values, characterizing interfacial adsorption of the reaction products on Ce_2_(MoO_4_)_3_/MWCNT-CPE. Furthermore, increasing the scan rate (25–250 mV s^−1^), there was a non-linear increase in *I_p_* and *E_p_* values (Figure 5B), which is typical of irreversible electrochemical reactions with product adsorption [41]. The effect of scan rate on reaction potentials is also useful for estimating the number of electrons in the reaction under study by the following equation:

(2) $$\left|E_{p}-E_{p/2}\right|=\frac{48}{\alpha n}$$

**Scheme 1:**
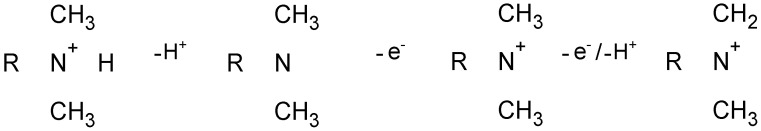
Mechanistic proposal for the electrooxidation of AZM on Ce_2_(MoO_4_)_3_/MWCNT-CPE.

Knowing that α represents the electron transfer coefficient and that irreversible oxidation of organic compounds usually results in α = 0.5, it is estimated that AZM loses 2*e*^−^ during the anodic process. The peak potential recorded in an alkaline medium corresponds to that expected for the oxidation of the amine group in the desosamine ring [42]. In this case, AZM oxidation begins with the deprotonation of the nitrogen atom (Scheme 1), followed by the loss of 1*e*^−^ and formation of a radical cation:

The radical cation is very reactive and quickly transfers one more *e*^−^, converting it to an enamine derivative. Apparently, its dimerization on the electrodic surface is moderately weak since the simple mechanical stirring of the electrolyte before measurements was enough to remove it and recover the initial current value. This is advantageous in electroanalytical systems because it minimizes the need for electrode polishing, which modifies the active area and impairs reproducibility.

The reaction mechanism and, consequently, the reactivity of the molecule can change depending on pH. In this work, the proton-dependent character of AZM was studied in a wide pH range (3–10), considering values lower and higher than its theoretical ionization constant (p*Ka* ≈ 8.5). Cyclic voltammograms in each condition can be seen in Figure 5C. For pH < 7, only low-intensity oxidation and reduction peaks are detected, indicating lower reactivity of the protonated molecule [41]. AT pH ≥ 7, a larger and well-defined oxidation peak is observed between 0.7 V and 1.0 V, being more suitable for electroanalytical purposes. Following intensity and reproducibility criteria, pH = 8 was used as the ideal condition in the remaining experiments.

### 3.4. Electroanalytical Parameters

Before AZM electroanalysis, the SWV current components were preliminarily evaluated using *f* = 100 Hz, *a* = 40 mV, and Δ*E_s_* = 5 mV. Figure S3 shows that the net component has greater intensity, since it is obtained from the sum of the forward and backward components, thus being more appropriate for developing the electroanalytical method. Regarding optimization of pulse frequency (*f*; 25–300 Hz), there was a linear increase in *I_p_* up to *f* = 100 Hz, as well as a displacement of *E_p_* and *E_p/_*_2_ towards more positive values. Since the SWV scan rate is directly affected by *f*, it is believed that the observed effects are also related to moderately slow AZM oxidation kinetics. The variation in pulse amplitude (*a*; 5–50 mV) provided a linear increase in *I_p_* values up to *a* = 40 mV, besides a succinct reduction in *E_p_* values. The potential increment (Δ*E_s_*; 2–7 mV) had a similar effect on the currents, but the opposite behavior for the *E_p_* values. The square wave voltammograms recorded under the different conditions mentioned above are compiled in Figure S4. Aiming to add greater sensitivity to the electroanalytical method, the following conditions were selected to construct the analytical curve: *f* = 100 Hz, *a* = 40 mV, and Δ*E_s_* = 5 mV.

**Figure 6:**
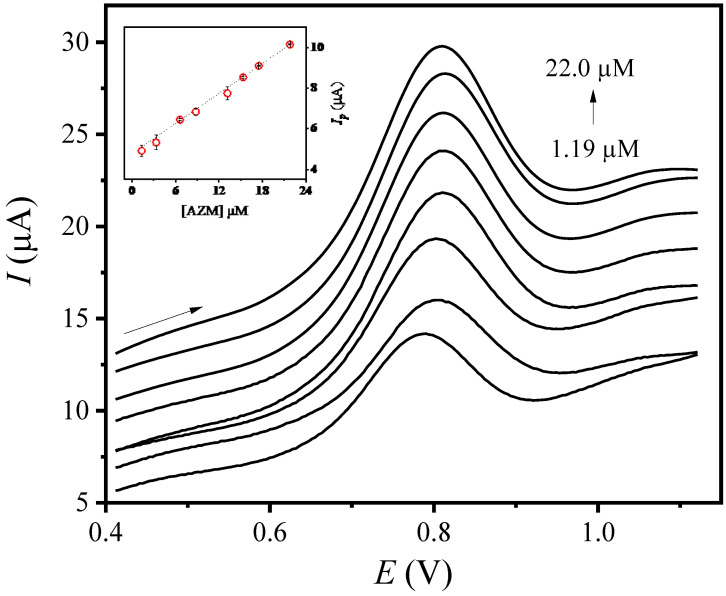
Square wave voltammograms recorded for AZM (1.19–22.0 µM) with the thermoactivated Ce_2_(MoO_4_)_3_/MWCNT-CPE sensor immersed in 1.0 mM phosphate buffer (pH = 8.0) and applying *f* = 100 Hz, *a* = 40 mV, and Δ*E_s_* = 5 mV. The resulting analytical curve is shown as an inset.

The electroanalytical performance of the thermoactivated Ce_2_(MoO_4_)_3_-MWCNT/CPE sensor to detect and quantify AZM in drug samples was evaluated by the linear regression coefficients resulting from the variation in analyte concentration (1.19–22.0 µM) and the corresponding *I_p_* values, as indicated in Figure 6. The working dynamic range showed high linearity and low dispersion of measurements (*R*^2^ = 0.9954; insertion in Figure 6), indicating that the following mathematical expression is statistically adequate to describe the data variance:

(3) $$I_{p}\left(\mu A\right)=5{.03\times 10}^{-6}\;(\mu A)+0.279\;(\mu A/\mu M)\;\left[\mathrm{AZT}\;(\mu M)\right]$$

The LOD and LOQ values were 230 nM and 750 nM, respectively, reaching levels as low as those sought for matrices of environmental concern (LOD ≈ 500 µM; [43]), body fluids (LOD ≥ 0.10 µM; [44]), cells, and pharmaceutical formulations (LOD ≥ 0.76 µM; [45,46]). This sensitivity was also better or similar to those found with other electrode materials, i.e., cobalt oxide (LOD = 925 nM; [47]), graphene (LOD = 303 nM; [42]) and cobalt ferrite–nickel oxide composites (LOD = 660 nM; [48]).

The intraday (*n* = 10) and interday (*n* = 5) repeatability tests performed with the same electrode also indicated low variance in the results (RDS ≤ 3.0%), as indicated in Table 1. Even using different devices (*n* = 3), only a subtle increase in data dispersion was noted, reiterating the credibility of the method. Additionally, these values were compared to those obtained by a reference spectrophotometric method [31], and the equivalence between them confirms the accuracy and reliability of the results. Excipients conventionally used in AZM-based formulations (lactose, polycaprolactone, poloxamer, and soy phospholipid) were also tested as possible interferers, but there was no influence on the electroanalytical signal, even at equimolar concentrations of the studied antibiotic.

### 3.5. AZM Thermal-Oxidative Stability

Given the performance of the thermoactivated Ce_2_(MoO_4_)_3_/MWCNT-CPE sensor for AZM electroanalysis, it was used to monitor the thermal-oxidative stability of the antibiotic at different temperatures (20–80 °C), times of exposure to UV radiation (1–3 h of irradiation at 257 nm), and air saturations (1–3 h of saturation with atmospheric air at 1.2 L min^−1^ and 15 kPa). The experiments conducted with each isolated parameter proved that, in fact, they all contribute to degrading the active ingredient (Figure S5), providing favorable conditions for chemical oxidation, although with different reaction kinetics. The thermal-oxidative degradation rate observed for the different variables follows a pseudo-second order model, with kinetic constants ranging from 0.006 to 0.042 M^−1^ s^−1^. When tested separately, the increase in temperature and saturation with atmospheric air had a greater impact on the process, reducing the AZM concentration to 40–78% of the initial value under the most critical conditions. The degradation of AZM when exposed to varying conditions of temperature and UV radiation or in contact with oxidizing species was also confirmed by other authors, through bioassays with *Bacillus subtilis* (ATCC 9372) [49] and UV spectrophotometry at 208 nm [50], strengthening the arguments presented.

Assuming that, in real conditions, these parameters (temperature—*A*, UV radiation—*B*, and air saturation—*C*) influence the stability of AZM collectively, and their combined effect was also verified by a 2^3^ full factorial experimental design, described by the following polynomial equation:

(4) $$\begin{array}{c} AZM{\;}_{degradation}\left(\%\right)=-93.03+3.56\;A+29.46\;B+0.63\;C-0.03\;A^{2}-11.88\;B^{2}\\ -0.01C^{2}+0.20\;AB-0.01\;AC+0.39\;BC \end{array}$$

**Figure 7:**
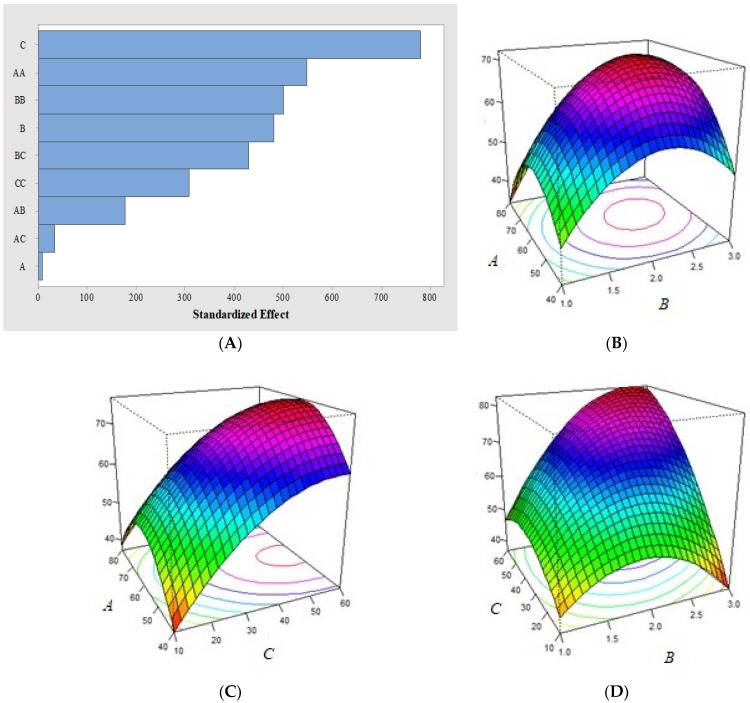
(**A**) Pareto chart and (**B**–**D**) Response surface plots obtained according to the significance level observed for the combined effect of temperature—*A*, UV radiation—*B*, and exposure to atmospheric air—*C* during the thermal-oxidative degradation of 10 µM AZM. The closer the color gets to the margin, the more critical the degradation is.

The adequacy of the analysis of variance (ANOVA; Table 2) for 95% confidence was confirmed by the Fisher’s *F*-test (*p* < 0.05) and correlation coefficient (*R*^2^ = 0.999) [32]. According to this model, parameter *A* has anomalous behavior if analyzed individually (*p* > 0.05), but it should not be disregarded since its quadratic and associated interactions reach *p* < 0.05, which proves synergy between the variables studied. According to the Pareto chart (Figure 7A), the following order of influence is established during the oxidation of AZM: *A* < *AC* < *AB* < *C*^2^ < *BC* < *B* < *B*^2^ < *A*^2^ < *C*. Evaluating the *AB* combination from the RSM plot (Figure 7B), the greatest degradation occurs around 60 °C and with 2.5 h of UV radiation. Higher temperatures reduce the oxygen concentration in the system, which may negatively interfere with the oxidation kinetics. For the AC combination (Figure 7C), the most representative effect occurs at 60 °C and after 1.0 h of saturation with atmospheric air, but it is less critical than BC with 3 h of UV radiation (induces electron transfer and photocleavage of covalent bonds [51]) and 1.0 h of sample oxygenation (Figure 7D). The most pronounced AZM degradation was recorded with *A* = 60 °C, *B* = 3 h, and *C* = 1 h, reducing the initial concentration by more than 81%. The RSM plots still show signs of thermal-oxidative degradation of AZM even under milder conditions, reinforcing the importance of stricter quality control to guarantee its stability and effectiveness.

## 4. Conclusions

The stability of the AZM molecule is affected by thermal-oxidative conditions different from those idealized in a laboratory environment; therefore, quality control of pharmaceutical formulations enriched with this active ingredient is strongly recommended. However, its analysis is not trivial, and the theoretical–experimental evidence obtained in this research revealed an unusual dependence on the solubility/electroactivity relationship in CH_3_OH/H_2_O, which controls the diversity of intermolecular interactions and solute solvation during measurements. In an alkaline electrolyte and after thermoactivation of Ce_2_(MoO_4_)_3_/MWCNT-CPE, this electrochemical sensor demonstrated a very attractive analytical performance to detect AZM with the necessary accuracy and reliability, even at submicromolar levels. Under thermal-oxidative conditions controlled by temperature, UV radiation, and saturation with atmospheric air, Ce_2_(MoO_4_)_3_-MWCNT/CPE was useful to monitor the accelerated degradation of AZM, becoming an alternative for laboratory routines focused on the effectiveness of pharmaceutical formulations and consumers’ health.

## Figures and Tables

**Table 1:** Repeatability and reproducibility tests performed with 5.00 µM AZM using the electroanalytical developed method and the reference spectroanalytical methodology [31].

Procedure	Intraday Repeatability (µM)	Mean (µM)	RSD (%)	Interday Repeatability (µM)	Mean (µM)	RSD (%)	Reproducibility (µM)	Mean (µM)	RSD (%)
Electroanalytical	5.05	4.97	1.5	5.05	4.91	2.8	5.05	4.98	3.1
	4.93			4.83			5.14		
	4.97			4.79			4.75		
	4.80			5.12					
	4.79			4.78					
	4.98								
	5.08								
	5.06								
	5.05								
	4.96								
Spectroanalytical	4.93	4.99	2.5	4.93	4.95	3.0	4.93	4.97	1.7
	5.10			4.76			5.09		
	5.12			5.15			4.88		
	4.84			5.12					
	4.83			4.78					
	5.08								
	5.05								
	5.15								
	4.87								
	4.81								

**Table 2:** ANOVA results obtained with the thermoactivated Ce_2_(MoO_4_)_3_/MWCNT-CPE sensor to evaluate the thermal-oxidative stability of AZM in 1.0 mM phosphate buffer (pH = 8.0) under different conditions of temperature (*A*), time of exposure to (*B*) UV radiation, and (*C*) saturation with atmospheric air.

Source	DF ^1^	SS ^2^	MS ^3^	*F*-Value	*p*-Value
Model	9	3.37 × 10^3^	3.74 × 10^2^	1.81 × 10^5^	1.48 × 10^−14^
Linear	3	1.74 × 10^3^	5.80 × 10^2^	281 × 10^5^	1.75 × 10^−13^
*A*	1	0.01	0.01	1.96	0.22
*B*	1	4.80 × 10^2^	4.80 × 10^2^	2.33 × 10^5^	7.28 × 10^−13^
*C*	1	1.26 × 10^3^	1.26 × 10^3^	6.09 × 10^5^	6.55 × 10^−14^
Quadratic	3	1.18 × 10^3^	3.92 × 10^2^	1.90 × 10^5^	5.13 × 10^−12^
*A* ^2^	1	6.25 × 10^2^	6.25 × 10^2^	3.02 × 10^5^	3.78 × 10^−13^
*B* ^2^	1	5.20 × 10^2^	5.20 × 10^2^	2.52 × 10^5^	5.97 × 10^−13^
*C* ^2^	1	1.95 × 10^2^	1.95 × 10^2^	9.48 × 10^4^	6.87 × 10^−12^
Two-factor interaction	3	4.50 × 10^2^	1.50 × 10^2^	7.27 × 10^4^	4.65 × 10^−13^
*AB*	1	65.0	65.0	3.14 × 10^4^	1.08 × 10^−10^
*AC*	1	2.37	2.37	1.15 × 10^3^	4.22 × 10^−7^
*BC*	1	3.83 × 10^2^	3.84 × 10^2^	1.85 × 10^5^	1.28 × 10^−12^
Lack of fit	3	<0.01	<0.01	0.15	0.923
Pure error	2	0.01	–	–	–
Total (model + residual)	14	3.37 × 10^3^	–	–	–
*R* ^2^	0.999	–	–	–	–

^1^ Degree of freedom. ^2^ Sum of squares. ^3^ Mean square.

## Data Availability

Data are contained within the article and Supplementary Materials.

## Supplementary Materials

The following supporting information can be downloaded at: https://www.mdpi.com/article/10.3390/nano14110899/s1, Table S1: Central composite design, predicted and observed AZM degradation values, starting with 10 µM of the antibiotic, obtained with the thermoactivated Ce_2_(MoO_4_)_3_/MWCNT-CPE sensor in 1.0 mM phosphate buffer (pH = 8.0). Square wave voltammetric conditions: *f* = 100 Hz, *a* = 40 mV, and Δ*Es* = 5 mV; Figure S1: X-ray diffraction pattern of Ce_2_(MoO_4_)_3_ used in photoelectrochemical sensor development; Table S2: Lattice parameters and quality indicators obtained by Rietveld refinement for Ce_2_(MoO_4_)_3_ samples; Figure S2: Cyclic voltammograms recorded in the absence and presence of 10 µM AZM with the thermoactivated Ce_2_(MoO_4_)_3_/MWCNT-CPE sensor at 50 mV s^−1^, using 1.0 mM phosphate buffer (pH = 8.0) prepared in CH_3_OH/H_2_O (10:90%, *v*/*v*) as electrolyte; Figure S3: Square wave voltammetry current components recorded for 10 µM AZM with the thermoactivated Ce_2_(MoO_4_)_3_/MWCNT-CPE sensor, using 1.0 mM phosphate buffer (pH = 8.0) prepared in CH_3_OH/H_2_O (10:90%, *v*/*v*) as electrolyte, and applying *f* = 100 Hz, *a* = 40 mV and Δ*E_s_* = 5 mV; Figure S4: Baseline-normalized square wave voltammograms recorded for 10 µM AZM with the thermoactivated Ce_2_(MoO_4_)_3_/MWCNT-CPE sensor under different conditions of (A) frequency, (B) amplitude, and (C) potential increment, using 1.0 mM phosphate buffer (pH = 8.0) prepared in CH_3_OH/H_2_O (10:90%, *v*/*v*) as electrolyte. During the optimization of each parameter, *f* = 100 Hz, *a* = 40 mV, and Δ*E_s_* = 5 mV were used; Figure S5: Percentage degradation of 10 µM AZM as a function of (A) temperature, exposure time to (B) UV radiation, and (C) saturation with atmospheric air.
