# Supplementary material for: Exploring the Thermal-Oxidative Stability of Azithromycin Using a Thermoactivated Sensor Based on Cerium Molybdate and Multi-Walled Carbon Nanotubes
Source: Nanomaterials (Basel). 2024 May 21;14(11):899. doi: 10.3390/nano14110899 (PMC11173558; doi:10.3390/nano14110899)
Supplement: Supplementary file 1 [file nanomaterials-14-00899-s001.zip › nanomaterials-2983191-supplementary.pdf]

Supplementary Material

Exploring the Thermal-Oxidative Stability of Azithromycin Using a Thermoactivated Sensor Based on Cerium Molybdate and Multi-Walled Carbon Nanotubes

Heryka R. A. Costa [1], André O. Santos [1], Yago N. Teixeira [1], Maria A. S. Silva [1], Valker A. Feitosa [2], Simone Morais [3] and Thiago M. B. F. Oliveira [1,*]

[1] Centro de Ciência e Tecnologia, Universidade Federal do Cariri, Av. Tenente Raimundo Rocha, 1639, Cidade Universitária, Juazeiro do Norte 63048-080, CE, Brazil; heryka.abrantes@aluno.ufca.edu.br (H.R.A.C.); andre.oliveira@ufca.edu.br (A.O.S.); yago.neco@aluno.ufca.edu.br (Y.N.T.); aparecida.santiago@ufca.edu.br (M.A.S.S.)

[2] Departamento de Tecnologia Bioquímico-Farmacêutica, Universidade de São Paulo, Av. Prof. Lineu Prestes, 580, Butantã, São Paulo 05508-000, SP, Brazil; valker@usp.br

[3] REQUIMTE–LAQV, Instituto Superior de Engenharia do Porto, Instituto Politécnico do Porto, Rua Dr. Bernardino de Almeida 431, Porto 4249-015, Portugal; sbm@isep.ipp.pt

* Correspondence: thiago.mielle@ufca.edu.br

Table S1. Central composite design, predicted and observed AZM degradation values, starting with 10 µM of the antibiotic, obtained with the thermoactivated $Ce_2(MoO_4)_3$/MWCNT-CPE sensor in 1.0 mM phosphate buffer (pH = 8.0). Square-wave voltammetric conditions: f = 100 Hz, a = 40 mV and ΔE_s = 5 mV.

Experimental conditions			AZM degradation (%)		*Relative error (%)*
A[1]	*B*[2]	*C*[3]	Predicted	Observed	
40	2	10	38.43	37.02	0.02%
60	3	10	36.90	36.91	0.02%
80	2	10	38.61	38.53	0.01%
80	3	35	57.57	54.30	0.02%
60	1	60	46.50	39.21	0.02%
60	2	35	70.65	67,59	0.09%
60	1	10	40.98	40.78	0.05%
40	2	60	63.65	63.59	0.01%
40	3	35	49.47	42.79	0.03%
80	1	35	34.01	34.02	0.04%
60	2	35	70.65	67.59	0.09%
60	2	35	70.65	70.61	0.09%
80	2	60	62.16	60.78	0.02%
60	3	60	81.59	81.61	0.02%
40	1	35	42.02	45.36	0.03%

[1] *Temperature.*
[2] *UV irradiation time.*
[3] *Saturation time with atmospheric air.*

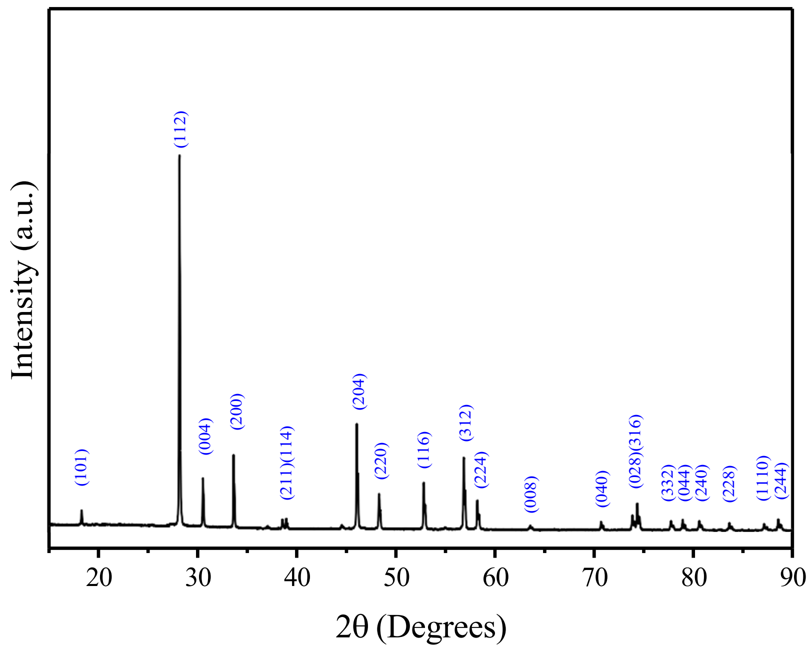

Figure S1. X-ray diffraction pattern of $Ce_2(MoO_4)_3$ used in photoelectrochemical sensor development.

Table S2. Lattice parameters and quality indicators obtained by Rietveld refinement for $Ce_2(MoO_4)_3$ samples.

Lattice parameter	*$Ce_2(MoO_4)_3$*	*ICSD - 423509*
a (Å)	5.325	5.331
c (Å)	11.725	11.830
c/a	2.203	2.219
V(Å^3)	332.23	336.300
χ^2	1.69	–
R_{exp} (%)	3.74	–
R_{wp} (%)	3.78	–
RF^2 (%)	10.11	–

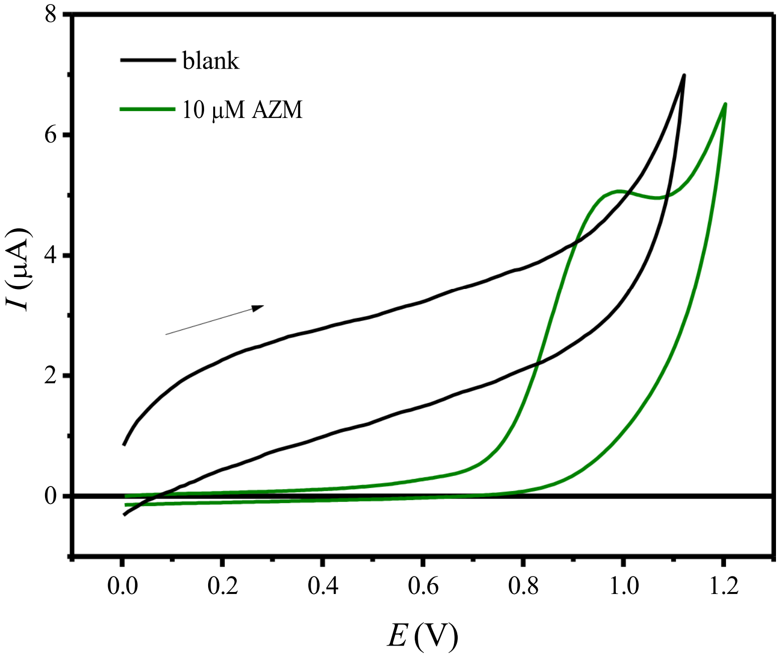

Figure S2. Cyclic voltammograms recorded in the absence and presence of 10 μM AZM with the thermoactivated $Ce_2(MoO_4)_3$/MWCNT-CPE sensor at 50 mV s^{-1}, using 1.0 mM phosphate buffer (pH = 8.0) prepared in CH_3OH/H_2O (10:90%, v/v) as electrolyte.

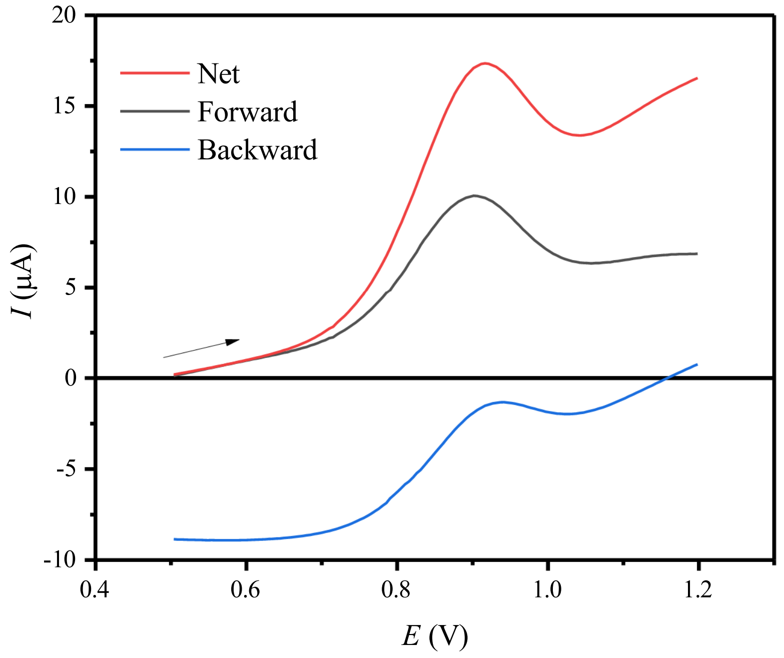

Figure S3. Square-wave voltammetry current components recorded for 10 μM AZM with the thermoactivated $Ce_2(MoO_4)_3$/MWCNT-CPE sensor, using 1.0 mM phosphate buffer (pH = 8.0) prepared in CH_3OH/H_2O (10:90%, v/v) as electrolyte, and applying f = 100 Hz, a = 40 mV and ΔE_s = 5 mV.

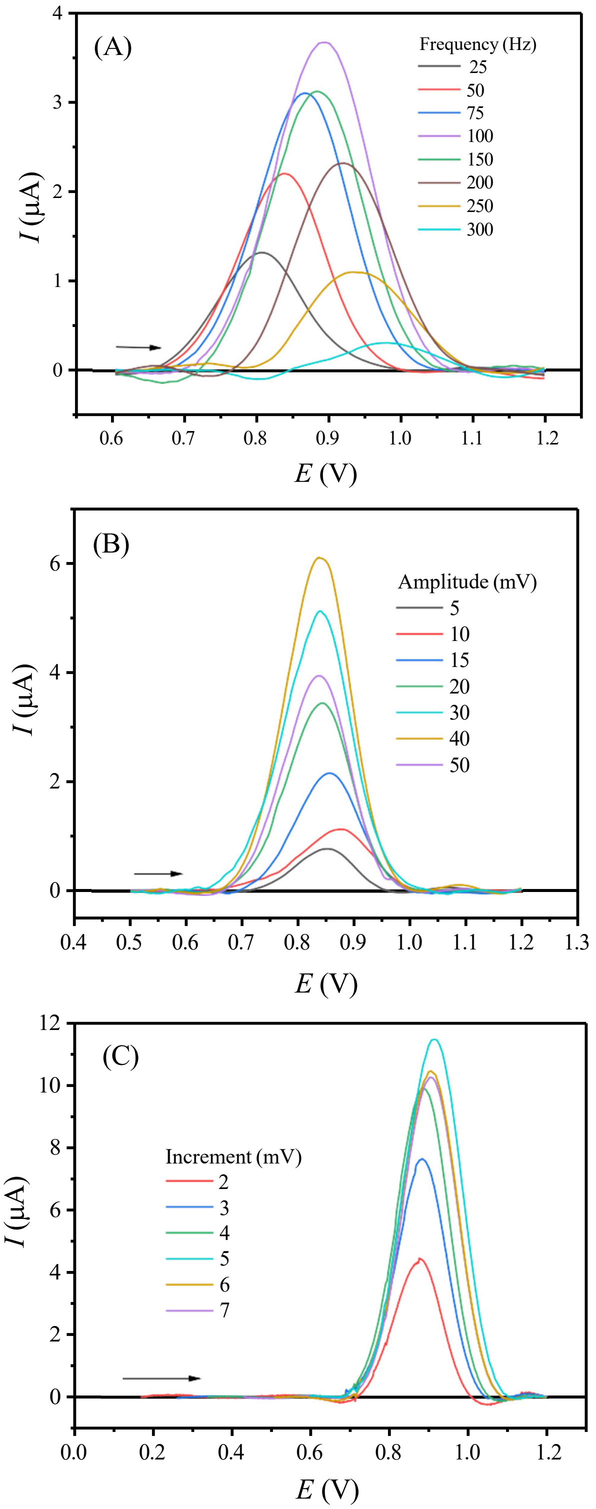

Figure S4. Baseline-normalized square-wave voltammograms recorded for 10 μM AZM with the thermoactivated $Ce_2(MoO_4)_3$/MWCNT-CPE sensor under different conditions of **(A)** frequency, **(B)** amplitude and **(C)** potential increment, using 1.0 mM phosphate buffer (pH = 8.0) prepared in CH_3OH/H_2O (10:90%, v/v) as electrolyte. During the optimization of each parameter, f = 100 Hz, a = 40 mV and ΔE_s = 5 mV were used.

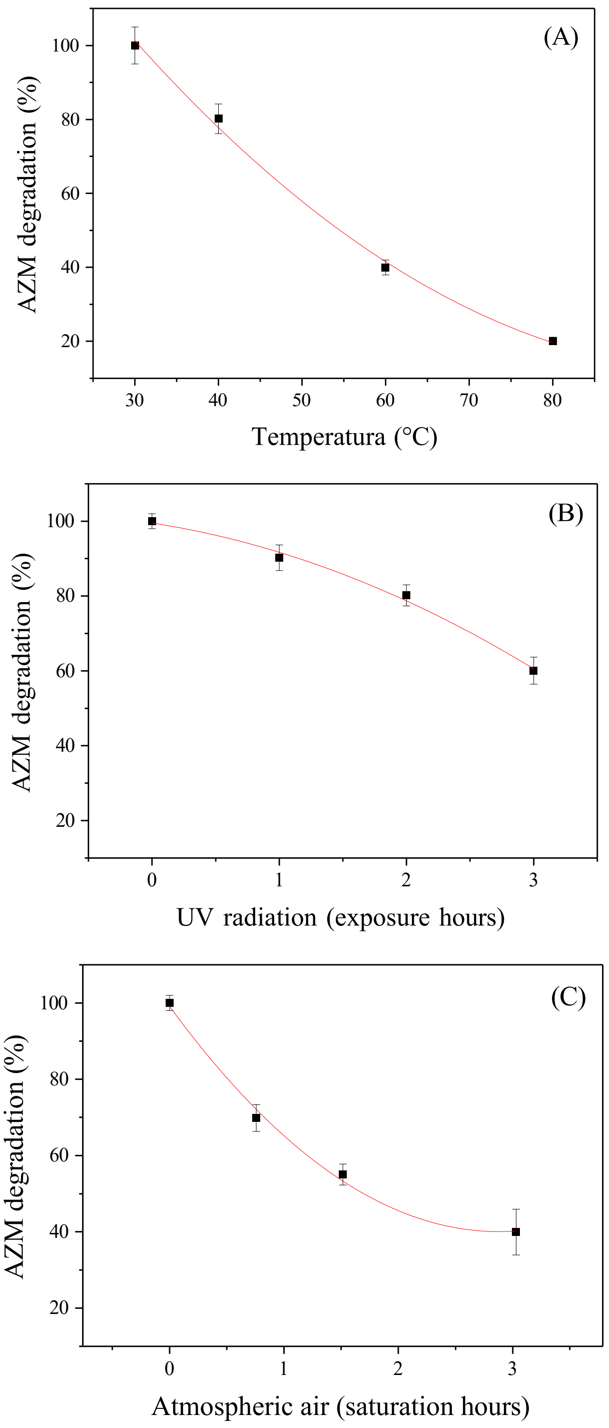

Figure S5. Percentage degradation of 10 μM AZM as a function of **(A)** temperature, exposure time to **(B)** UV radiation and **(C)** saturation with atmospheric air.
